# Acute pancreatitis associated with severe acute respiratory syndrome coronavirus-2 infection: a case report and review of the literature

**DOI:** 10.1186/s13256-021-03026-7

**Published:** 2021-09-09

**Authors:** Abdullah S. Eldaly, Ayman R. Fath, Sarah M. Mashaly, Muhammed Elhadi

**Affiliations:** 1 Plastic and Reconstructive Surgery Department, Tanta University Hospital, Tanta, Egypt; 2 Internal Medicine Department, Creighton University Arizona Health Education Alliance, Phoenix, AZ USA; 3 Elmenshawy General Hospital, Tanta, Egypt; 4 Faculty of Medicine, University of Tripoli, University Road, Furnaj, 13275 Tripoli, Libya

**Keywords:** Acute pancreatitis, Pancreas, SARS-CoV-2, COVID-19, Case report

## Abstract

**Introduction:**

We report a case of Severe acute respiratory syndrome coronavirus-2 infection with acute pancreatitis as the only presenting symptom. To the best of our knowledge, there are few case reports of the same presentation.

**Case presentation:**

An otherwise healthy 44-year-old white male from Egypt presented to the hospital with severe epigastric pain and over ten attacks of nonprojectile vomiting (first, gastric content, then bilious). Acute pancreatitis was suspected and confirmed by serum amylase, serum lipase, and computed tomography scan that showed mild diffuse enlargement of the pancreas. The patient did not have any risk factor for acute pancreatitis, and extensive investigations did not reveal a clear etiology. Given a potential occupational exposure, a nasopharyngeal swab for polymerase chain reaction testing for severe acute respiratory syndrome coronavirus 2 was done, which was positive despite the absence of the typical symptoms of severe acute respiratory syndrome coronavirus 2 such as fever and respiratory symptoms. The patient was managed conservatively. For pancreatitis, he was kept *nil per os* for 2 days and received intravenous lactated Ringer’s (10 ml per kg per hour), nalbuphine, alpha chymotrypsin, omeprazole, and cyclizine lactate. For severe acute respiratory syndrome coronavirus 2, he received a 5-day course of intravenous azithromycin (500 mg per day). He improved quickly and was discharged by the fifth day. We know that abdominal pain is not a rare symptom of severe acute respiratory syndrome coronavirus 2, and we also know that elevated levels of serum amylase and lipase were reported in severe acute respiratory syndrome coronavirus-2 patients, especially those with severe symptoms. However, the association between severe acute respiratory syndrome coronavirus-2 infection and idiopathic acute pancreatitis is rare and has been reported only a few times.

**Conclusion:**

We believe further studies should be conducted to determine the extent of pancreatic involvement in severe acute respiratory syndrome coronavirus-2 patients and the possible causality between severe acute respiratory syndrome coronavirus 2 and acute pancreatitis. We reviewed the literature regarding the association between severe acute respiratory syndrome coronavirus 2 and acute pancreatitis patients. Published data suggest that severe acute respiratory syndrome coronavirus 2 possibly could be a risk factor for acute pancreatitis.

## Introduction

With over 149 million confirmed cases and 3.14 million deaths worldwide as of 29 April 2021, coronavirus disease 2019 (COVID-19) has declared itself the most significant global health emergency humanity had to face in decades [1]. After more than 10 months of the pandemic, we still lack a comprehensive understanding of the virus pathophysiology and how it manifests in different patients. Gastrointestinal (GI) manifestations were reported in about 18% of patients, with diarrhea being the most commonly reported GI symptom [2] that is most likely due to alteration of enterocyte permeability [3]. Mild-to-moderate liver injury was reported as well, and the exact mechanism is still not fully understood [3]. Acute abdominal pain has also been reported, and its exact pathophysiology is still elusive. Acute pancreatitis was reported a few times as a cause of abdominal pain in patients with severe acute respiratory syndrome coronavirus 2 (SARS-CoV-2), and it is not clear if the virus could involve the pancreas specifically. We are reporting a case of COVID-19 presenting with acute pancreatitis without other risk factors for pancreatitis.

## Case presentation

A previously healthy 44-year-old white male presented to the emergency department with severe epigastric pain radiating to the back and frequent vomiting (over ten attacks, first gastric content, then bilious with no blood) for 3 days on 22 June 2020. Four days before the beginning of his abdominal symptoms, the patient received a laboratory diagnosis of severe acute respiratory syndrome coronavirus 2 (SARS-CoV-2) after undergoing a nasopharyngeal swab for reverse-transcription polymerase chain reaction (RT-PCR) to detect SARS-CoV-2 infection as part of surveillance screening after contacting several COVID-19 patients during his work in a hotel in Sharm El-Sheikh, Egypt, and the patient was asked to self-isolate. However, he presented to our care after 5 days of SARS-COV-2 diagnosis with acute pancreatitis. The patient did not have any respiratory symptoms at that time.

During history taking, the patient denied any respiratory symptoms such as cough or dyspnea. The patient denied smoking, alcohol, or drug intake (illicit or therapeutic except for occasional use of paracetamol for right knee pain). The patient was a middle-class worker who denied exposure to any hazardous substances at his work place. He also denied any previous similar attacks of abdominal pain. There was no family history of similar attacks. His vital signs were as follows: blood pressure of 94/50 mmHg, pulse rate of 112 beats per minute, respiratory rate of 27 breaths per minute, temperature of 37.5 °C, and oxygen saturation of 98% on room air. Abdominal examination revealed marked tenderness in the epigastric region without distension. Neurological examination did not reveal any abnormalities. Intravenous fluid resuscitation was started immediately with a bolus of 1.5 L of lactated Ringer’s.

A repeat nasopharyngeal Reverse transcription polymerase chain reaction (RT-PCR) was performed on the day of admission to the hospital as a part of the local protocols for suspected cases. The results came back positive on 24 June 2020. The patient was then transferred from the medical ward to an isolation center in the same hospital, explicitly dedicated to COVID-19 patients.

**Fig. 1:**
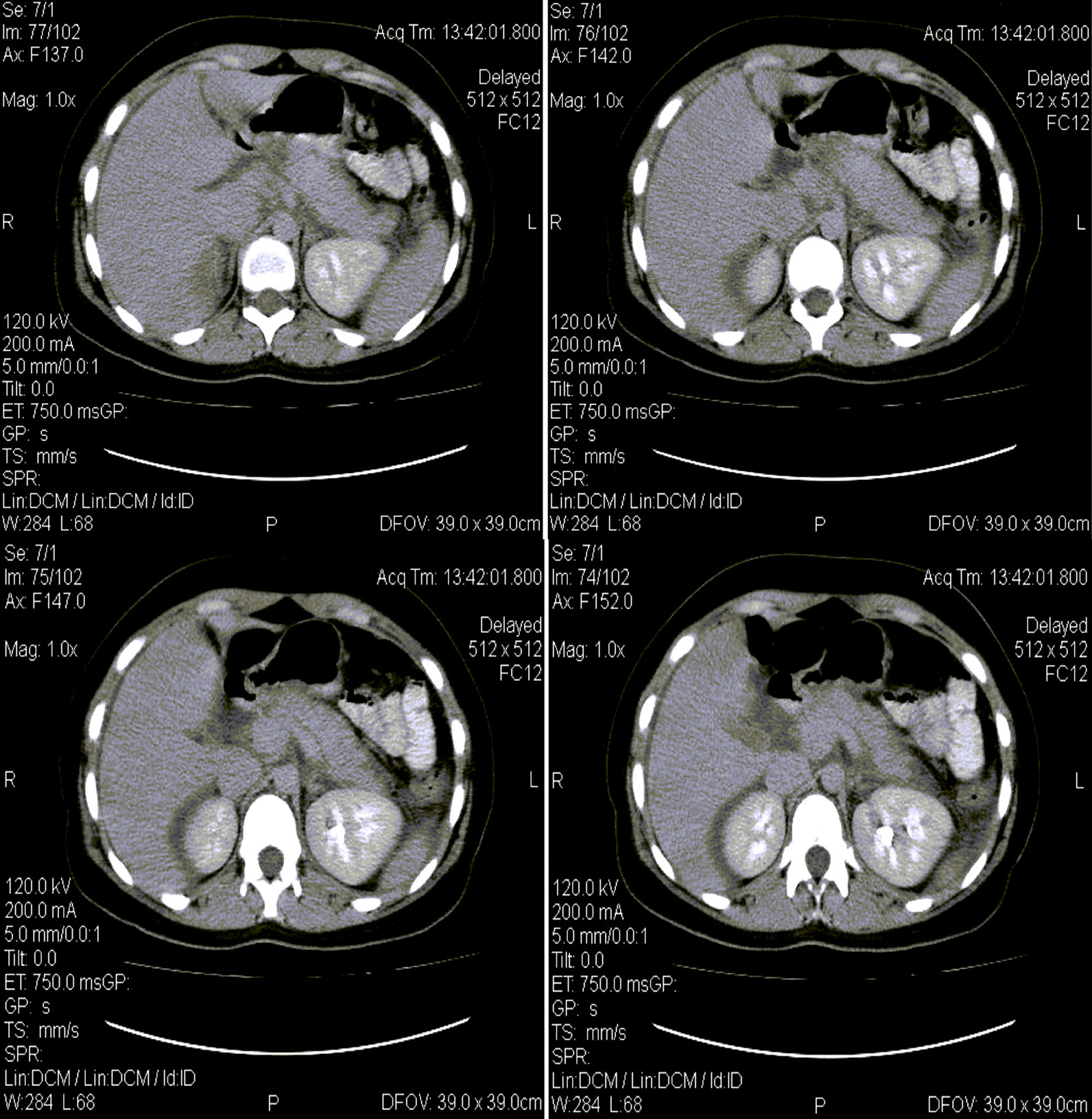
Computed tomography scan of the abdomen showing diffuse enlargement of the pancreas

An abdominal X-ray was done and was unremarkable. However, abdominal–pelvic ultrasonography revealed mild diffuse enlargement of the pancreatic head with normal gall bladder and biliary tract. Serum amylase was 773, and serum lipase was 286 (Table 1). The diagnosis of mild acute pancreatitis was confirmed with an abdominal CT scan that revealed mild diffuse enlargement of the pancreas (Figure 1). The patient was managed conservatively for 4 days. For pancreatitis, he was kept NPO for 2 days during which he received 10 ml/kg/hour of lactated Ringer’s; he also received nalbuphine (10–20 mg per day, intramuscular) for analgesia, omeprazole (40 mg once daily, intravenous) for ulcer prophylaxis and cyclizine lactate (50 mg twice daily, intravenous) for vomiting. On the third day, anorexia and vomiting subsided, and the patient was started on oral feeding, which he tolerated well. On the fifth day, the patient was vitally stable and symptom-free and was advised to continue his SARS-CoV-2 treatment from home, including ascorbic acid (1 g per day, oral) and zinc sulfate (220 mg per day, oral). Abdominal ultrasound was repeated on discharge and again showed no gallstones. At 30 days follow-up, the patient was well and did not have any complaints.

**Table 1:** Laboratory results on admission

Laboratory results		Normal range
White cell count (per mm^3^) WBC differential (per mm^3^)	17,700	4000–11,000
Total neutrophils	13,140	2500–8000
Total lymphocytes	2850	1000–4000
Total monocytes	910	100–700
Platelet count (per mm^3^)	386,000	1,47,000–3,47,000
Alanine aminotransferase (U/L)	176	29–33
Aspartate aminotransferase (U/L)	158	5–40
Total bilirubin (mg/dl)	1	0.1–1.2
Direct bilirubin (mg/dl)	0.2	Less than 0.3
Lactate dehydrogenase (U/L)	222	140–280
Blood urea nitrogen (mg/dl)	39	7–20
Creatinine (μmol/L)	1.07	0.8–1.2
Amylase (U/L)	773	30–110
Lipase (U/L)	286	0-160
Triglycerides (mg/dl)	119	Less than 150
Total cholesterol (mg/dl)	221	Less than 200
C-reactive protein (mg/L)	38	Less than 6
Random blood glucose (mg/dl)	151	80–140
Hepatitis A virus serology	Negative	
Hepatitis B virus serology	Negative	
Hepatitis C virus serology	Negative	
Human immunodeficiency virus serology	Negative

## Discussion

Although rare, acute pancreatitis can be caused by viral, bacterial, fungal, and parasitic infections. Viral pancreatitis is known to be caused by mumps, cytomegalovirus, hepatitis B virus, herpes simplex virus, varicella-zoster virus, and human immunodeficiency virus (HIV) [4–6]. Although coronaviruses are not known to cause pancreatitis in humans, the 2003 SARS was associated with damage to the endocrine pancreas and acute diabetes [7]. This effect was explained by damage to acinar cells through the virus binding to angiotensin-converting enzyme 2 (ACE2) receptors [7].

Liu *et al*. reported elevated amylase and lipase in 16.41% and 1.85% of patients with severe and mild SARS-CoV-2 infections, respectively, suggesting some degree of pancreatic injury [8]. This injury’s exact pathophysiology is not well understood, but SARS-CoV-2 may involve the exocrine pancreas in the same manner SARS involves the endocrine pancreas: through ACE2 receptor binding, especially now that we know that SARS-CoV-2 binds ACE2 receptors ten times stronger than the 2003 SARS [9]. A recent study published by Müller *et al*. found that SARS-CoV-2 has the ability to infect and replicates in β-cell of pancreatic islets of Langerhans as they detected SARS-CoV-2 nucleocapsid protein in the pancreatic cells of postmortem patients [10]. Their findings may explain the reason behind the metabolic dysregulations of COVID-19 patients, such as impaired glucose homeostasis and abnormal amylase or lipase levels [8].

We report the first African case report of acute pancreatitis presenting as SARS-CoV-2 infection. Our patient had acute acalculous pancreatitis in association with SARS-CoV-2 infection. We managed to exclude alcoholism, drugs, hypertriglyceridemia, hypercalcemia (by laboratory testing), and trauma (by history) as potential etiologies. The patient denied any previous attacks or family history of similar symptoms. We did not test our patient for autoimmune pancreatitis since this was not feasible at our institution. Also, we did not test for viral causes of pancreatitis other than hepatitis B virus and human immunodeficiency virus, which both were negative.

We searched the literature in PubMed/Medline up to 3 January 2021 to identify published case reports of COVID-19 associated with pancreatitis. We identified only 29 cases published in 25 articles (Table 2). SARS-CoV-2 infections were diagnosed with RT-PCR in all cases except one case with antibody testing. Three cases were in the pediatric age group < 18 years. Including our case, patients have a mean age of 43.5 years, and 14 were males (46.6%). The majority of the cases had abdominal pain and/or vomiting, 82% of patients had elevated serum lipase, and almost all patients had elevated serum lipase and/or amylase. Moreover, 72% of patients had abdominal CT findings suggestive of pancreatitis. All patients were discharged alive, except two patients were still in the intensive care unit (ICU), and only one patient died (Table 3).

**Table 2:** Published cases of acute pancreatitis associated with SARS-CoV-2 infection

Author	Country	Age	Sex	Pulmonary symptoms	Extra-pulmonary symptoms	Physical examination findings	Chest CT	Abdominal CT	Serum lipase and serum amylase	SARS-CoV-2 RT-PCR	Other laboratory test performed to exclude other etiologies	Outcome
Myeres *et al*. [13]	USA	67	Male	Acute hypoxic respiratory failure	Acute onset epigastric abdominal discomfort and fever	Epigastric tenderness	Ground-glass opacity in the right lung apex	Acute interstitial edematous pancreatitis with moderate peripancreatic stranding and edema	L: 5295 U/L A: not reported	Not done; only SARS-CoV-2 rapid test was positive 3 days after onset of abdominal pain and 2 days after hospitalization	Liver chemistry tests, serum triglycerides, serum immunoglobulin G4	Alive
Samies *et al*. [14]	USA	15	Male	Nasal congestion	Anosmia, ageusia, vomiting, and abdominal pain	Epigastric tenderness	Scattered ground-glass opacities in bilateral lung fields	Mild stranding around the head of the pancreas	L: 233 U/L (4–39 U/L) A: not reported	Positive 2 days after onset of abdominal pain and 1 day after hospitalization	Liver chemistry tests, serum triglycerides.	Alive
Samies *et al*. [14]	USA	11	Male	None reported	Headache, chills, tactile fever, abdominal pain, hematochezia, and epistaxis	Epigastric tenderness	Interstitial opacities with peribronchial thickening	Fatty infiltration of the liver, enlarged appendix, and normal pancreas	L: 582 U/L (4–39 U/L) A: 156 U/L	Positive on the same day of onset of abdominal pain and 2 days prior to hospitalization	Liver chemistry tests, serum triglycerides (elevated to 251 mg/dl), cholesterol (normal)	Alive
Samies *et al*. [14]	USA	16	Female	Cough	Subjective fever, nausea, and abdominal pain	Epigastric tenderness	Not evaluated	Hepatomegaly, single gallstone, and prominence of the pancreas	L: 1909 U/L (4–39 U/L) A: not reported	Positive 1 week prior to onset of abdominal pain	Liver chemistry tests, serum triglycerides, cholesterol	Alive
Fernandes *et al*. [15]	Brazil	36	Female	Dyspnea	Fever, headache, and abdominal pain	Not reported	Bilateral pulmonary opacities	Acute interstitial pancreatitis with acute peripancreatic fluid collection	L: 640 U/L A: 710 U/L	Positive	None reported	Alive
Lakshmanan *et al*. [16]	USA	68	Male	None reported	Loss of appetite, anorexia, nausea, and vomiting	Dehydration, lethargy, and soft, nontender abdomen	Not evaluated	Peripancreatic fat stranding, most remarkable around the tail, with mild duodenal wall thickening and adjacent fat stranding, likely from pancreatitis. The gallbladder appeared normal, without wall thickening or surrounding inflammatory changes, and the common bile duct was not dilated	L: 2035 U/L A: 1030 U/L	Positive 2 days prior to hospitalization and 7 days prior to diagnosis of pancreatitis	Liver chemistry tests, total bilirubin, serum triglycerides, serum calcium	Alive
Kurihara *et al*. [17]	Japan	55	Male	Severe respiratory distress necessitated intubation and ECMO	Could not be evaluated due to sedation	Could not be evaluated due to sedation	Not evaluated	Pancreas with diffuse parenchymal enlargement and stranding of the surrounding retroperitoneal fat	L: 263 U/L (16–55 U/L) A: 252 U/L (44–132 U/L)	Positive on day 8 after respiratory symptom onset	Serum triglycerides (mild elevation), serum calcium	Alive
Alves *et al*. [18]	Brazil	56	Female	Dry cough and dyspnea	General malaise and epigastric pain	Not reported	Multiple ground-glass opacities, interlobular septal thickening, and consolidation areas	Heterogeneously enhancing and edematous pancreas	L: 2993 U/L A: 544 U/L	Positive	Serum triglycerides (209 mg/dl), serum calcium (1.24 mg/dl)	Alive
Wang *et al*. [19]	China	42	Male	Chest discomfort and shortness of breath	Nausea and persistent upper abdominal pain with radiation to the back for 3 days	Not reported	Multiple ground-glass opacities in both lungs	The prominence of the pancreas and peripancreatic fluid accumulation, without biliary dilatation or microlithiasis	L: 382 U/L (0–180 U/L) A: 132 U/L (0–180)	Positive on day 5 of abdominal pain	Serum triglycerides: 3.2 mmol/L ( < 1.7 mmol/L), Serum calcium	Dead
Wang *et a*l. [19]	China	35	Male	None reported	Five days of persistent upper abdominal pain with radiation to the back, nausea, and vomiting	Not reported	Patchy shadows in the lower right lung and bilateral pleural effusion	Pancreatic swelling, peripancreatic fluid accumulation, and prerenal fascial thickening without biliary dilatation or microlithiasis	L: 1042 U/L (0–180 U/L) A: normal	Positive on day 6 of abdominal pain	Serum triglycerides: 3.97 mmol/l ( < 1.7 mmol/l), Serum calcium	Alive
Patnaik *et al*. [20]	India	29	Male	Dyspnea	Acute diffused abdominal pain of 5 days duration that radiated to the back and progressively worsened and low-grade fever	Abdominal tenderness maximal in the umbilical region	Not evaluated	Swollen pancreas	L: 1650 U/L A: 2861 U/L	Positive	Serum triglycerides, serum calcium	Alive
Kumaran *et al*. [21]	UK	67	Female	None reported	Epigastric pain, diarrhea, and vomiting	Not reported	Not evaluated	Necrotizing pancreatitis	L: not evaluated A: 1483 U/L	Positive	Liver chemistry tests, serum triglycerides, serum calcium, immunoglobulin G4	Alive
Gonzalo-Voltas *et al*. [22]	Spain	76	Female	None reported	Epigastric pain, fever, vomiting, and diarrhea	Not reported	Not evaluated	Interstitial edematous pancreatitis	L: not evaluated A: 3568 IU/L	Positive	None reported	Alive
Cheung *et al*. [23]	USA	38	Male	None reported	Fever and epigastric pain	Not reported	Not evaluated	Not evaluated	L: 10,255 ukat/L	Positive 1 week prior to presenting in the emergency department	Liver chemistry tests, serum triglycerides, serum calcium, serum bilirubin	Alive
Kataria *et al*. [24]	USA	49	Female	Dry cough, shortness of breath, and hypoxic respiratory failure	Fever, nausea, vomiting, and severe abdominal pain radiating to the back	Not reported	Multifocal infiltrates involving the posterior basal segment of the left lower lobe and an apical–posterior segment of the left upper lobe	Diffuse enlargement of pancreas with ill-defined borders and surrounding peripancreatic fluid	L: 1451 IU/L (0–160) A: 501 IU/L (30–110)	Positive on the second day of hospitalization	Liver chemistry tests, serum triglycerides, serum cholesterol, serum calcium, total bilirubin	Alive
Brikman* et al*. [25]	Israel	61	Male	Cough, dyspnea, and hypoxemia	Fever, weight loss, and diffuse abdominal tenderness	Soft abdomen with no signs of peritoneal irritation	Not evaluated	Focal parenchymal enhancement of the pancreas head with inflammatory changes in peripancreatic fat	L: 203 U/L (21–67 U/L) A: 142 U/L (28–100 U/L)	Positive	Serum triglycerides: 3.18 mmol/L (1.8 mmol/L), direct bilirubin	Alive
Mazrouei *et al*. [26]	UAE	24	Male	Mild upper respiratory tract symptoms	Nonradiating epigastric pain, nausea, and vomiting	Epigastric discomfort on palpation	Not evaluated	Edema of the distal pancreas with surrounding fluid	L: 578 IU/L A: 391 U/L	Positive 1 day prior to presenting to the emergency department	None reported	Alive
Bokhari *et al*. [27]	Pakistan	32	Male	Sore throat and productive cough	High fever, chills, severe epigastric pain radiating to back, and nonbilious vomiting	Not reported	Not evaluated	Bulky and swollen pancreas with significant peripancreatic inflammatory changes and fluid collection along the gastrosplenic ligament	L: 721 IU/L A: 672 IU/L	Positive 8 days after onset of symptoms.	Liver chemistry tests, serum triglycerides, serum calcium	Alive
Alloway *et al*. [28]	USA	7	Female	None reported	Fever and abdominal pain	Distension and tenderness to palpation in the left upper and left lower quadrant, and the epigastric regions	Not evaluated in the second attack (the first attack showed small bilateral pleural effusion)	Not evaluated in the second attack (the first attack showed necrotizing pancreatitis)	L: 676 U/L in the first attack 1672 U/L in the second attack (80–360 U/L) A: not reported	Not done in the first attack Positive in the second attack	Serum LDH	Alive
Rabice *et al*. [29]	USA	36	Female (33 weeks pregnant)	Dry cough and dyspnea	Nausea, vomiting, and epigastric pain	Epigastric tenderness	Not evaluated	Not evaluated	L: 875 U/L A: 88 U/L	Positive	Liver chemistry tests. Serum triglycerides (210 mg/dl)	Alive
Pinte *et al*. [30]	Romania	47	Male	Dry cough	Severe epigastric pain with radiation to the back, nausea, constipation, and lack of flatus	Epigastric tenderness	Scattered bilateral subpleural ground- glass opacities	Blurring of the pancreatic contours due to the edema of the surrounding adipose tissue	L: 22× upper limit of normal A: 6× upper limit of normal	Positive	Serum triglycerides, serum calcium, gamma-glutamyltranspeptidase	Alive
Meireles *et al*. [31]	Portugal	36	Female	Dry cough, breathlessness, and fever	Nausea, vomiting, and epigastric pain	No physical findings	Bilateral ground-glass opacities with 75–100% lung involvement	No pancreatic abnormalities	L: 631 U/L A: 718 U/L	Positive 4 days after onset of cough	Serum triglycerides, serum cholesterol, serum calcium, ANA screening. Anti-HIV 1 and 2, HBs antigen, anti-HCV antibody, anti-Coxsackie antibody (IgM/IgG), anti-herpes virus 1 antibody (IgM/IgG), anti-herpes virus 2 antibody (IgM/IgG), anti-CMV antibody (IgM/IgG)	Alive
Miao *et al*. [32]	France	26	Female	None reported	Fever, epigastric pain, and severe vomiting	Not reported	Bilateral basal condensations and pleural effusions	Enlarged pancreas gland without any structural abnormality	L: 211 U/L A: not reported	Positive	Liver chemistry tests, serum triglycerides, serum calcium, serological tests for human immunodeficiency virus, hepatitis B and C, Coxsackie viruses, *Chlamydia*, *Mycoplasma*, antinuclear and anti-DNA antibodies	Alive
Aloysius *et al*. [33]	USA	36	Female	Dry cough and progressive dyspnea	Fever, stabbing epigastric pain, vomiting, and diarrhea	Severe epigastric tenderness	Multifocal bilateral ground-glass opacities	Normal	L: 627 U/L A: 325 U/L	Positive	Liver chemistry test, serum triglycerides, serum procalcitonin, total and direct bilirubin	Unknown
Hadi *et al*. [34]	Denmark	47	Female	Acute respiratory distress	None reported	Not reported	Not evaluated	Not evaluated	L: not evaluated A: more than 1500 U/L	Positive	Serum triglycerides, serum calcium	Still in ICU
Hadi *et al*. [34]	Denmark	68	Female	Dyspnea and hypoxia that necessitated intubation and mechanical ventilation	Abdominal pain	Epigastric tenderness	Not evaluated	Not evaluated	L: not evaluated A: 934 U/L	Positive	Serum triglycerides, serum calcium	Still in ICU
Anand *et al*. [35]	UK	59	Female	Cough and sore throat	Fever, myalgia, abdominal pain, and constipation	Not reported	Not evaluated	A previously atrophic pancreas that had increased markedly in size and had features of diffuse edematous changes, suspicious for acute pancreatitis	L: not evaluated A: not evaluated	Positive	None reported	Alive
Hassani *et al*. [36]	Iran	78	Female	None reported	Severe positional epigastric pain precipitated by lying supine, nausea, vomiting, and chills with no fever	Epigastric tenderness	Patchy peripheral ground glass infiltrations in both lungs	Not evaluated	L: 230 IU/L A: 185 IU/L	Positive	Liver chemistry tests, lipids profile, serum electrolytes	Alive
Kandasamy *et al*. [37]	India	45	Female	None reported	Severe sharp epigastric pain radiating to the back	Severe epigastric tenderness	Multifocal areas of ground-glass opacities, consistent with CO-RADS score of 5	Diffusely enlarged pancreas with acute peripancreatic and pararenal collections	L: 293 IU/L A: 364 IU/L	Positive	Liver chemistry, total bilirubin, gamma-glutamyltransferase, alkaline phosphatase	Alive

L, serum lipase; A, serum amylase.

**Table 3:** Summary of the previous case reports important statistics

	Percentage of patients (%)
Abdominal pain	89
Vomiting	45
Elevated serum lipase	82
Elevated serum amylase	69
Elevated serum lipase and/or amylase	100
CT evidence of pancreatitis	72
Discharged alive	86

Acute pancreatitis appears to be an infrequent complication/association of COVID-19. One retrospective study from the USA analyzing 11,883 patients with COVID-19 found that the point prevalence of pancreatitis was 0.27% (32 patients) [11]. However, another prospective international study of acute pancreatitis during the COVID-19 pandemic concluded that acute pancreatitis with SARS-CoV-2 infection has a higher risk of severity and poor clinical outcomes, including the risk of organ dysfunction higher 30-day inpatient mortality compared with acute pancreatitis patients who are SARS-CoV-2-negative [12].

## Conclusion

Until solid evidence on the relation between pancreatitis and SARS-CoV-2 is provided, we believe acute pancreatitis should be considered a potential explanation for acute abdominal pain in SARS-CoV-2 patients. Such evidence should rise from well-designed epidemiological studies as well as autopsy studies.

## Data Availability

All data and reports are present upon request.

## Abbreviations

SARS-COV-2: Severe acute respiratory syndrome coronavirus 2
RT-PCR: Reverse-transcription polymerase chain reaction
NPO: *Nil per os*
